# Impact of a *Gap Junction Protein Alpha 4* Variant on Clinical Disease Phenotype in *F508del* Homozygous Patients With Cystic Fibrosis

**DOI:** 10.3389/fgene.2020.570403

**Published:** 2020-10-28

**Authors:** Tabea Horn, Michael Ludwig, Olaf Eickmeier, Anne H. Neerinex, Anke H. Maitland-van der Zee, Christina Smaczny, Thomas O. F. Wagner, Ralf Schubert, Stefan Zielen, Christof Majoor, Lieuwe D. Bos, Sabina Schmitt-Grohé

**Affiliations:** ^1^Abt. Allgemeine Pädiatrie, Zentrum für Kinderheilkunde des Universitätsklinikums Bonn, Bonn, Germany; ^2^Institut für Klinische Chemie und Klinische Pharmakologie des Universitätsklinikums Bonn, Bonn, Germany; ^3^Department for Children and Adolescents, Division of Allergology, Pulmonology and Cystic Fibrosis, Goethe University, Frankfurt, Germany; ^4^Department of Respiratory Medicine, Amsterdam University Medical Centers, University of Amsterdam, Amsterdam, Netherlands; ^5^Department of Pediatric Respiratory Medicine, Amsterdam UMC, University of Amsterdam, Amsterdam, Netherlands; ^6^Christiane-Herzog CF-Ambulanz, Universitätsklinikum Frankfurt, Frankfurt, Germany; ^7^Department of Intensive Care Medicine, Amsterdam UMC, University of Amsterdam, Amsterdam, Netherlands

**Keywords:** cystic fibrosis, *F508del* homozygous, lung disease phenotype, lung function, inflammatory markers, phenotype/genotype relation, precision medicine, *gap junction protein alpha 4*-genotype

## Abstract

**Background:**

Lung disease phenotype varies widely even in the *F508del* (homozygous) genotype. Leukocyte-driven inflammation is important for pulmonary disease pathogenesis in cystic fibrosis (CF). Blood cytokines correlate negatively with pulmonary function *in F508del* homozygous patients, and gap junction proteins (GJA) might be related to the influx of blood cells into the lung and influence disease course. We aimed to assess the relationship between *GJA1/GJA4* genotypes and the clinical disease phenotype.

**Methods:**

One-hundred-and-sixteen homozygous *F508del* patients (mean age 27 years, m/f 66/50) were recruited from the CF centers of Bonn, Frankfurt, and Amsterdam. Sequence analysis was performed for *GJA1* and *GJA4*. The clinical disease course was assessed over 3 years using pulmonary function tests, body mass index, *Pseudomonas aeruginosa* colonization, diabetes mellitus, survival to end-stage lung disease, blood and sputum inflammatory markers.

**Results:**

Sequence analysis revealed one clinically relevant single nucleotide polymorphism. In this *GJA4* variant (rs41266431), homozygous G variant carriers (*n* = 84/116; 72.4%) had poorer pulmonary function (FVC% pred: mean 78/86, *p* < 0.040) and survival to end-stage lung disease was lower (*p* < 0.029). The frequency of *P. aeruginosa* colonization was not influenced by the genotype, but in those chronically colonized, those with the G/G genotype had reduced pulmonary function (FVC% pred: mean 67/80, *p* < 0.049). Serum interleukin-8 (median: 12.4/6.7 pg/ml, *p* < 0.052) and sputum leukocytes (2305/437.5 pg/ml, *p* < 0.025) were higher for the G/G genotype.

**Conclusions:**

In carriers of the A allele (27.6%) the *GJA4* variant is associated with significantly better protection against end-stage lung disease and superior pulmonary function test results in *F508del* homozygous patients. This SNP has the potential of a modifier gene for phenotyping severity of CF lung disease, in addition to the *CFTR* genotype.

**Clinical Trial Registration:**

The study was registered with ClinicalTrials.gov, number NCT04242420, retrospectively on January 24th, 2020.

## Introduction

Progressive pulmonary destruction is the major cause of morbidity and mortality in cystic fibrosis (CF) patients (Cantin et al., 2015). The most important factor in the pathogenesis of CF lung disease is leukocyte-driven inflammation. Blood cytokines correlate negatively with pulmonary function in *F508del* homozygous patients (Schmitt-Grohé et al., 2005; Eickmeier et al., 2013). Although several reports have found an association between *F508del* and pancreatic insufficiency (Johansen et al., 1991), there is variability how it affects lung disease phenotype (Cystic Fibrosis Genotype-Phenotype Consortium, 1993; McKone et al., 2006).

It has been suggested that genetic variation in modifier genes contributes more to the expression of the pulmonary phenotype than does the *Cystic Fibrosis Transmembrane Conductance regulator (CFTR) protein* variant (Cutting, 2015). In his review on monozygotic twins (in comparison to dizygotic twins), Cutting reported a maximal 25% impact of the *CFTR* genotype on lung disease (Cutting, 2015).

The question arises what kind of molecular mechanisms influence the degree of the ongoing neutrophilic inflammation in CF lung disease in addition to the CFTR dysfunction. Lung inflammation is caused by the CF basic defect and secondary reactions due to microbial pathogens. Here the airway epithelia act as a biological physical barrier. Key components of this barrier are not only tight junctions but also gap junctions proteins (called connexins as well).

Gap junction proteins (GJA) or connexins (Cx) are a family of transmembrane proteins, which oligomerize into hexameric structures to form a hemichannel (connexon). Connexons of both cells can align and dock to form a gap junction channel: Gap junction intercellular communication (GJIC) confers a continuous link between the cytoplasm of both cells (Saab et al., 2014). They allow the passage of inorganic ions and of small water soluble molecules; thus, coupling the cells both electrically and metabolically (Saab et al., 2014). In addition, connexons were shown to provide communication pathways or GJIC, for small molecules between the intracellular and extracellular environments. For instance, connexons have been proposed as a putative channel for ATP release (Saab et al., 2014). GJAs regulate mucociliary clearance by coordinating ciliary beating, surfactant secretion, airway surface liquid (ASL) volume and mucus secretion. The human GJA family comprises 21 members, with GJA1 being the most frequently expressed in tissues.

Gap junction protein A1 (GJA1) (=Connexin 43), encoded by the *GJA1* gene has been detected in human airway epithelial cells (Losa and Chanson, 2015). Moreover in terms of neutrophil recruitment, GJA1 promotes their transmigration across the alveolar wall during the acute phase of inflammation (Losa and Chanson, 2015). There are some specific features for GJA1 in CF: Downregulation of *GJA1* expression in cell culture models (Carbone et al., 2018) showed that GIJC may regulate *CFTR* expression and function that modulate airway epithelium tightness. Moreover Molina and coworkers (Molina et al., 2015) were able to provide evidence that CF airway epithelial cells display a lower transepithelial resistance (TER) and a GJA1 mistracking to the plasma membrane as compared with wild-type cells. This suggests that a defect in gap junctions might influence TER. In addition the defective regulation of GJA1 in CF airway epithelial cells may contribute to the reduced apoptosis and bacterial killing of *P. aeruginosa* in CF (Losa and Chanson, 2015).

Gap junction protein A4 (GJA4) (=connexin 37), encoded by the *GJA4* gene, is expressed in human bronchiolar, alveolar epithelial cells and in pulmonary artery endothelial cells (Freund-Michel et al., 2016). Moreover there is evidence of GJA 4 on macrophages (Valdebenito et al., 2018) and neutrophils (Sáez et al., 2014). In inflammatory diseases, GJA4 hemichannels (present in primary monocytes and macrophages) may regulate monocyte adhesion to the endothelium (Valdebenito et al., 2018). In sepsis increased Nitric oxid (NO) production targets GJA 4. This results in vasodilation due to an impaired arteriolar-conducted vasoconstriction mediated by GJA 4 as key player (McKinnon et al., 2009; Tyml, 2011). This vasodilation may slow down blood flow, increasing adhesion of macrophages and neutrophils, facilitating diapedesis of blood cells. The role of GJA 4 in microvascular dysfunction as a systemic inflammatory response may extend to other chronic inflammatory diseases like CF.

In summary, so far there is evidence of *GJA4* and *GJA1 (Cx37 and Cx43)* expression on airway epithelial cells, neutrophils and macrophages as well as vascular endothelium in humans (Freund-Michel et al., 2016). Pulmonary disease in CF is dominated by a leukocyte driven inflammation. GJAs might be of importance for the influx of blood cells into the lung. This might contribute to neutrophilic inflammation and long term prognosis in CF.

In this regard, our hypothesis was that *GJA4* or *GJA1* genotypes have an impact on clinical disease phenotype in F508del homozygous CF patients. To our knowledge, *GJA4* and *GJA1* variants have not been studied in human beings with CF yet. This is the first report of this genetic association with a replication effort as well.

## Materials and Methods

### Patients

A total of 116 *F508del* homozygous patients (66 male and 50 female Caucasian subjects (*p* < 0.739), 98 German and 18 Dutch patients) from the 3 CF centers participated. The initial cohort was from the University Hospital of Bonn (*n* = 26). For replication patients from Frankfurt (*n* = 72) and Amsterdam UMC (*n* = 18) were recruited. The mean age was 27 years (confidence interval [CI], 24.6–29.4, range 7–61). Microbiological testing results were available for 115 patients.

The exclusion criteria were treatment with systemic steroids 14 days preceding this trial, participation in another study within the past 30 days, treatment with Orkambi, and lung transplantation (for assessment of lung function). The protocol was approved by the Ethics Committee of the Universities of Bonn (178/01 + 092/17), Frankfurt (07/02 + 206/16), and Amsterdam UMC (NL60220.018.16). Informed consent was obtained from all patients or from their parents. The study was registered with ClinicalTrials.gov (NCT04242420), retrospectively on January 24th, 2020.

All subjects underwent spirometry (forced expiratory volume in one second [FEV1], forced vital capacity [FVC], and forced expiratory flow at 75% of the pulmonary volume [FEF75]) and supplied a blood sample (ethylenediamine tetraacetic acid). Pulmonary function tests were performed according to the recommendations of the American Thoracic Society and the European Respiratory Society (Miller et al., 2005).

### Genotyping

For gene analysis, standard procedures were used for the isolation of genomic DNA, amplification of DNA via polymerase chain reaction (PCR), and performance of the automated sequencing analyses.

In brief, primers were directed to the single coding exons 2 of *GJA1* (=*gap junction protein alpha 1*) (GenBank acc.no. NM_000165; 2F 5′-CTTTTCTTCGTTGGCAAAAATGG-3′; 2R 5′- CGGATCAAAATTAACACCTGGTG-3′) and *GJA4* (=*gap junction protein alpha 4*) (GenBank acc. no.: NM_002060; 2F: 5′-AGACCTCCCTGCAGGCTTGT-3′; 2R: 5′-AGTCATCT CTGCAGAGCCTTCC-3′). The resultant PCR products were subjected to direct automated sequencing (3130XL Genetic Analyzer, Applied Biosystems, Foster City, CA, United States). For each patient, both strands of the amplicons were sequenced, and PCR primers served as sequencing primers. All analyses were performed in the genetic laboratory in Bonn (Institut für Klinische Chemie und Pharmakologie).

### Pulmonary Function Tests

Pulmonary function tests, i.e., FEV1, FVC, and maximum expiratory flow at 25% of the FVC (FEF75) were performed using a Master Screen Body or IOS (Vyaire Medical Gmbh, Würzburg, Germany), in Bonn and Frankfurt. Carefusion Jaeger Pneumo Vyntus was used in Amsterdam. As lung volume is dependent on height and age, pulmonary function data were presented as percent predicted (for height, sex, and age). To accurately assess individual lung function, the median pulmonary function test results over a 3-year period as a percent predicted of the global lung initiative (GLI) values were acquired for the German patients. The data were provided by using the German CF registry (MUKOWEB, www.mukoviszidose-register.de). For the Bonn cohort (step 1 registry data) only one value per year was available.

For the majority of the Frankfurt patients, step 2 registry data with more than one visit at the CF center per year were available. For these patients, the visit with the best pulmonary function data for FEV1 in a given year was selected. The median age was calculated as the second year of the 3 observation years. As the Dutch lung function data were cross-sectional (1 measurement for 2017 available) and the German data were longitudinal (if possible 2018–2016), the German cohort was analyzed separately.

### Inflammatory Markers

Functional studies were performed by assessing blood and sputum parameters: white blood cell count and interleukin-8 (IL-8). IL-8 in the serum was measured by chemiluminescence (Immulite, Siemens Healthcare Diagnostics, Eschborn, Germany, formerly DPC Biermann), as described in previous papers (Schmitt-Grohé et al., 2005; Eickmeier et al., 2010, 2013).

### Statistical Analysis

For statistical analysis after categorization into carriers and non-carriers (causing homozygosity for the G variant) of allele A (for *GJA 4* at position rs41266431), we compared the aggregated outcomes (median over 3 years) of continuous data. For parametric data, the mean, standard deviation, and 95% CIs were calculated and tested by student *t*-tests. Non-parametric data were presented as the median and interquartile range (IQR) and tested by the Mann-Whitney-U-Test for unpaired samples. Sputum culture results were used to categorize patients as *Pseudomonas aeruginosa* positive or negative. Chronic and intermittent colonization was defined according to the Leeds criteria (Lee et al., 2003). The binary data created were analyzed by Pearson’s chi-squared test. In addition, a mixed linear model was used to estimate the effect of the genotype and chronic *P. aeruginosa* colonization on pulmonary function. Additional multivariate analysis included covariates such as age and body mass index (BMI). Survival analysis was carried out by plotting Kaplan-Meier estimates, calculating log-rank-test, and fitting Cox proportional hazard models to the data. As lung transplantation was an exclusion criterion in terms of evaluation of pulmonary function, survival-to-end-stage CF was addressed separately. Of the entire cohort, there were six lung transplants with two patients still alive. Thus, 15 patients were classified as having end-stage CF (death or lung transplant) and were analyzed separately. A *p*-value of < 0.05 was accepted to indicate statistical significance. All calculations were performed using SPSS (Version 25.0).

## Results

Gene sequence data were available for all subjects. Although *GJA1* sequencing yielded only one heterozygous single nucleotide polymorphism (SNP) (rs138386744) in one patient, four common SNPs were detected after *GJA 4* sequencing. Two of these were synonymous changes, but the third (rs41266431: GTA valine, ATA isoleucine) and fourth (rs1764391: CCC proline, TCC serine) SNPs caused amino acid substitutions at protein positions 130 and 319, respectively. For SNP (rs41266431), no evidence for a deviation from Hardy-Weinberg equilibrium was detected (*p* = 0.2287). In the analysis of the initial cohort of the CF center Bonn, only this SNP revealed an impact on clinical course resp. lung function. In this regard only this SNP was evaluated in the replication cohorts of Frankfurt and Amsterdam.

For rs41266431, patients were grouped into those homozygous for the G allele (G/G genotype, *n* = 84, 72.4%) and carriers for the A allele (A/G + A/A genotype, *n* = 32, 27.6%). The allele and genotype frequencies for the three sub-cohorts are provided in Table 1.

**TABLE 1:** Frequency of alleles and genotypes for *gap junction protein alpha 4* at position *rs41266431* in 3 CF cohorts.

Cohort	Allele/Genotype	*N* (%)
Bonn	*n* = 26	
	G	0.827
	A	0.173
	A/G	9 (35)
	A/A	0 (0)
	Genotype (G/G)	17 (65)
	Genotype (A/G + AA)	9 (35)
Frankfurt	*n* = 72	
	G	0.89
	A	0.11
	A/G	14 (19)
	A/A	1 (1)
	Genotype (G/G)	57 (79)
	Genotype (A/G + A/A)	15 (21)
Amsterdam	*n* = 18	
	G	0.75
	A	0.25
	A/G	7 (39)
	A/A	1 (6)
	Genotype (G/G)	10 (56)
	Genotype (A/G + A/A)	8 (44)

Patient characteristics, as well as clinical parameters according to the *GJA4* genotype, are provided in Table 2.

**TABLE 2:** Patients characteristics.

	*rs41266431*	
	
	*Genotype G/G*	(*A/G + A/A*)
	(*n* = 84)	(*n* = 32)

	Mean	
Age (years)	26.5	28.4
Sex (m/f) (n)	(47/37)	(19/13)
*P. aeruginosa*+ (%)	64%	71%
BMI *(% pred.*)	29.7	38.6
Diabetes mellitus^1^	32%	29%
FEV1 *(% pred.)*	65.7	70.6
FEF75 *(% pred.*)	53.8	63.4
FVC (%predicted)	78.2^#^	86.2^#^

*m, male; f, female; *P. aeruginosa*+, *Pseudomonas aeruginosa* colonization (intermittent + chronic); FEV1 (% predicted), forced expiratory volume in 1 s in% predicted, FEF75 (% predicted) forced expiratory flow at 75% of the pulmonary volume (FEF75), FVC(% predicted) forced vital capacity in% predicted, ^#^*p* < 0.04, ^1^only Germans.*

### Mortality

There was no difference in the observation time for the two genotypes (G/G genotype/carriers of the A allele): mean 29.1/31.6 years, 95% CI [26.5–31.8, 26.5–36.7 years], range 9–58/12–62 years, *p* < 0.355). Among those without lung transplantation there were nine deaths homozygous for the G allele and no death among the carriers of the A allele (heterozygous as well as homozygous). The median age of death was 30 years. The log rank test (Kaplan-Meier curves) indicated that the carriers of the A allele survived longer (*p* < 0.044). There were no deaths in the Dutch cohort, and the log rank test for the German cohort revealed a trend for longer survival for the carriers of the A allele (genotype G/G/genotype A allele carrier: mean 29.5/33 years, 95% CI [26.5–32.5, 26.3–39.6 years], range 9–58/12–62 years, *p* < 0.053) as well.

Overall, up to 2018, a total of 13 patients who did (*n* = 4) or did not (*n* = 9) receive a lung transplant died. Thus, 13 patients with the G/G genotype and no carriers of the A allele (*p* < 0.009) died. As there were no deaths in the Dutch cohort, the log rank test for the German cohort revealed a significant difference (*p* < 0.013) as well.

**FIGURE 1:**
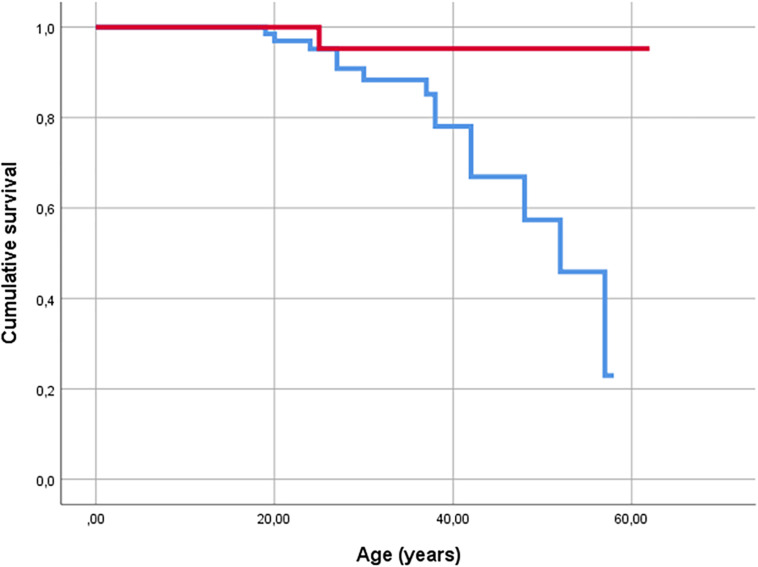
Kaplan-Meier plot of survival of CF patients. Events are defined as death (*n* = 13) and lung transplantation (*n* = 2) (end stage CF). Red line indicates carriers of A allele, and blue line indicates G/G homozygous patients.

End-stage lung disease: Fourteen patients had the G/G genotype, and one CF patient who was alive after lung transplantation was a carrier of the A allele. End-stage CF was significantly more common in those with G/G genotype *p* < 0.029) (Figure 1). As there were no end-stage CF patients in the Dutch cohort, the German cohort was analyzed separately (*p* < 0.039). Cox regression analysis revealed a seven-fold (CI 0.914–54.006) higher risk for those with the G/G genotype to experience severe lung disease (*p* < 0.061). For the German cohort, the risk was 6.5-fold (CI 0.84–49.9) higher for G/G genotype than for carriers of the A allele (*p* < 0.073).

As there were no deaths for the carriers of the A allele and 13 for in the G/G genotype, in patients aged ≥30, there was a significant difference in the distribution of age (genotype G/G/(A/G + A/A): mean 38.7/46.5 years, CI 36.32–41.2/40.1–51.9, *p* < 0.004). In this regard, we performed a matched pair analysis by age (± 2 years) and sex (Table 3).

**TABLE 3:** Characteristics of matched patients.

	*rs41266431*	
	
	*Genotype G/G*	*A/G + A/A*
	(*n* = 22)	(*n* = 22)

	Median	
Age (years)	29.5	27.5
Sex (m/f)	(14/8)	(14/8)
*P. aeruginosa (chronic)*	10/22	7/22
Diabetes mellitus	6/22	5/22
BMI (*percentile*)	27	25
FEV1 *(% predicted)*	48*	68*
FEF75 *(% predicted*)	20^§^	45.5^§^
FVC *(% predicted*)	65^&^	84^&^

*p < 0.097, ^§^*p* < 0.031, ^&^*p* < 0.012.

### Pulmonary Function

Spirometry data were available for all 116 patients for FEV1, 110 for FVC, and 111 for FEF75 (Table 2). In the German cohort, there also was a significant difference in the FVC (% predicted) between those with the G/G genotype and for the carriers of the A allele (mean 76/85.3, CI 72.7–83.3/78.3–92.4, *p* < 0.038) (see online data Supplementary Table 1).

The results of the mixed linear model (adjusted for age, BMI, and chronic colonization of *P. aeruginosa*) indicated a poorer FVC (% pred) (estimated –7.7%, CI (−15.3)–(−0.1), *p* < 0.047) for the G/G genotype. This was also the case for the German subcohort (estimated –9.8%, CI (−18.2)–(−1.3), *p* < 0.025).

### P. aeruginosa

Sixty-six (66%) patients had microbiological evidence of *P. aeruginosa* colonization (chronic *n* = 57 [49.6%], intermittent *n* = 19 [16.5%]). The *GJA4* genotype did not have an impact on general colonization (intermittent + chronic) (*p* < 0.502), or on chronic colonization (*p* < 0.268) in the overall cohort (*n* = 115) (Table 2). This was also the case for the German cohort (where longitudinal data were available); for subcohorts see the online data supplement. The characteristics of patients with chronic *P. aeruginosa* colonization are provided in Table 4.

**TABLE 4:** Characteristics of patients with chronic *P. aeruginosa* colonization.

	*rs41266431*	
	
	*Genotype G/G*	(*A/G + A/A*)
	(*n* = 39)	(*n* = 18)

	Mean	
Age (years)	32.7	35.6
Sex (m/f)	(26/13)	(12/6)
Diabetes mellitus	51.4%	38.5%
BMI (*percentile*)	24.1	25.9
FEV1 *(% predicted)*	51.1	62.1
FEF75 *(% predicted*)	37.8	51.5
FVC *(% predicted*)	67.4^&^	80^&^
Interleukin-8 pg/ml (Serum)^1^	9.35*	6.1*

^&^*p* < 0.049, *p < 0.033, ^1^median (*n* = 16/8).

To determine the impact of *P. aeruginosa* on the clinical course, longitudinal data (only available from the German cohort) were evaluated as well (Supplementary Table 2).

### Inflammatory Markers

Blood and sputum parameters were available for a subset of 29 German patients from former studies (Schmitt-Grohé et al., 2005; Eickmeier et al., 2010, 2013). Carriers of the A allele had lower IL-8 serum values (G/G genotype/(A/G + A/A): median 12.4/6.7 pg/ml, IQR 7.95–24.7/5.9–10.1, *p* < 0.052) and sputum leukocyte counts (G/G genotype/(A/G = A/A): median 2305/438 pg/ml, IQR 544.4-3400/20-617.5, *p* < 0.025) (Table 5).

**TABLE 5:** Functional data by genotype.

	*rs41266431*	
	
	*Genotype G/G*	(*A/G + A/A*)
	(*n* = 21)	(*n* = 8)

	Median	
Age (years)	18	16
Sex (m/f) (n)	(10/11)	(5/3)
*P. aeruginosa*+ (%)	43%	50%
FEV1 *(% pred.)*	66	86.5
FEF75 *(% pred.*)	36.15	41
VC (%predicted)	74.33	84.5
IL-8 pg/ml (Serum)	12.4*	6.7*
Leukocytes/μl (Sputum)	2305^#^	437.5^#^

m, male; f, female; *P. aeruginosa*+, *Pseudomonas aeruginosa* colonization (intermittent + chronic); FEV1 (% predicted), forced expiratory volume in 1 s in% predicted, FEF75 (% predicted) maximum expiratory flow at 75% of forced vital capacity, VC(% predicted) vital capacity in% predicted *p < 0.052, #*p* < 0.025.

## Discussion

The findings of this study provide evidence that one SNP, rs41266431, in *GJA4* causes an amino acid substitution with a pronounced clinical impact: carriers of the A (27.6%) allele have significantly better protection against end-stage lung disease and superior pulmonary function. This genotype has no impact on the frequency of *P. aeruginosa* colonization, but those who are carriers of the A allele and chronically colonized with *P. aeruginosa* have significantly better pulmonary function than the G/G genotype.

To our knowledge, this is the first study to report on this SNP in CF patients. In this regard, the epidemiologic and functional issues should be discussed: The allele frequencies of *GJA4* (= *Cx37)* at position rs41266431, as provided by United States National Center for Biotechnology Information^1^ for Europe (*A* = 0.13160/*G* = 0.86840) are in keeping with our findings for the overall cohort (*A* = 0.147/*G* = 0.853) and for the German cohort (*A* = 0.128/*G* = 0.872). Moreover in a sample of healthy German adults (*n* = 38) we found similar allele frequencies (*A* = 0.183/*G* = 0.817). The significant differences (*p* < 0.046) compared to the Dutch cohort (*A* = 0.25/*G* = 0.75) might be attributed to the small sample size.

It is unclear why this important SNP in *GJA 4* was not identified as a modifier in the genome-wide association study (GWAS) by Corvol et al. (2015), who also assessed lung function parameters over a 3-year period. One possible explanation might be that in the GWAS cohort no German or Dutch patients were included. Similar, the transforming growth factor beta SNP, which showed an excellent power by the Drumm study (Drumm et al., 2005), was not identified as significant in the GWAS study. However, there was overlap between the GWAS and Drumm cohorts.

The Val130Ile polymorphism was first reported by Krutovshikk (Krutovskihh et al., 1996), but its role in gating and conductance of gap junction channels has never been tested (Kumari et al., 2000). Our finding raises the question as to how the *GJA 4* p.Val130Ile conservative substitution may exert a protective effect. This amino acid position varies among species, but valine or isoleucine are the only residues found at the corresponding *GJA 4* position as far down as Xenopus. Because exonic sequence motifs can determine splicing patterns and amounts of transcript (Ohno et al., 2018), we used various tools to predict the possible effects of the exonic G-to-A substitution underlying the p.Val130Ile exchange. Whereas Human Splicing Finder (Human Splicing Finder, 2009; Desmet et al., 2009) scored the variation with most probably no impact on splicing (Supplementary Table 3), ESE Finder (ESE Finder, 2003; Cartegni et al., 2003) and SFMap (SFmap, 2010; Paz et al., 2010) predicted several binding motifs affected by rs41266431. Here, SFMap detected binding sites for serine/arginine-rich splicing factor SR30 (SF2ASF), heterogeneous nuclear ribonucleoproteins A2/B1 (hnRNPA2B1), and heterogeneous nuclear ribonucleoprotein H (hnRNPH1), all deleted by the presence of the A allele. ESE finder predicted a decreasing score for recognition of the motif for splicing factor SRSF1 and a higher score for binding of SRSF2 with the A-nucleotide in the respective motif. Hence, rs41266431 may modulate the efficiency of splicing exon 2 to the untranslated exon 1, thereby leading to different expression of *GJA4* mRNA. However, another as of yet unknown variant located in the vicinity and segregating with the A-allele may exert a protective effect.

To our knowledge, of the modifier genes, only mannose-binding lectin gene heterogeneity has been associated with survival in CF (Garred et al., 1999). In this regard, *GJA4* is the second modifier gene that exerts an impact on survival. It is striking that there were no deaths among the 32 carriers of the A allele with a mean observation time of 31.6 years (age range 12–62 years). The Cox regression analysis revealed a seven-fold higher risk of experiencing severe lung disease in patients with the G/G genotype. However, this was only a trend (*p* < 0.061), and respectively did not reach significance, which might be due to the sample size.

Others studied measures like survival to end stage CF (death or lung transplantation) (Garred et al., 1999). For this endpoint, we found a significant difference between the two genotypes. Interestingly, there were no deaths among the carriers of the A allele, but there was one lung transplantation at the age of 25 years. The reason for this transplant was extreme incompliance. If the G/G genotype is linked to higher mortality respectively end-stage CF, what clinical features are associated with it?

In the overall cohort, carriers of the A allele exhibited significantly better pulmonary function in terms of FVC (% predicted) than the G/G genotype. With increasing age, the chronic inflammatory reactions cause fibrotic remodeling of lung tissue and restrictive airway disease, presenting as reduced FVC. The matched pair analysis (Table 3) in the entire German cohort (Dutch excluded due to only cross-sectional data) for carriers of the A allele had significantly better FVC and FEF75 (% predicted), but there was only a trend for FEV1. This can be explained by the small sample size (*n* = 22 each group).

This SNP did not have an impact on the frequency of intermediate or chronic colonization with *P. aeruginosa*, but the G/G genotype was associated with more severe lung disease in CF patients with chronic *P. aeruginosa* colonization. *P. aeruginosa* induces IL-8 secretion. IL-8 is a chemoattractant for leukocytes in the lung. Our functional data revealed higher IL-8 levels and sputum leukocytes in patients with the G/G genotype. So far we cannot provide evidence for a functional link between the *GJA 4* genotype and IL-8 secretion, But the mechanisms discussed in the *in silico* analysis should be part of further investigations. In particular the influence of the A allele on splicing and consecutively *GJA4* mRNA expression should be elucidated. In this regard, the SNP might have an influence on IL-8 secretion and influx of leukocytes into the lung. We have provided evidence that the SNP at rs41266431 for *GJA4* has an influence on the severity of *P. aeruginosa* colonization-associated lung disease in *F508del* homozygous CF patients.

One potential limitation of this study is stratification. For the Bonn and Frankfurt CF centers, longitudinal data were available, and every patient fulfilling the inclusion criteria participated. As for the Amsterdam CF cohort, cross-sectional data were available for only 18 patients. In this regard, stratification might be an issue for the Amsterdam cohort. Thus, the German patients were analyzed separately, supporting the findings of the overall cohort.

## Conclusion

In summary, our data indicate significantly better protection against end-stage lung disease in carriers of the A allele (27.6%). This is associated with superior pulmonary function test results. The SNP has no influence on the frequency of *P. aeruginosa* colonization, but it has a protective effect on lung function in those chronically colonized with *P. aeruginosa*. Future research should be directed toward the mechanism how this SNP influences mRNA expression of *GJA4*, IL-8 secretion, influx of leukocytes into the lung. Therefore, this SNP has the potential of a modifier gene for phenotyping severity of CF lung disease, in addition to the *CFTR* genotype.

## Data Availability Statement

The datasets presented in this study can be found in online repositories. The names of the repository/repositories and accession number(s) can be found in the article/ Supplementary Material.

## Ethics Statement

The studies involving human participants were reviewed and approved by University of Bonn (178/01 + 092/17), University of Frankfurt (07/02 + 206/16), and Amsterdam University Medical Center (NL60220.018.16). Written informed consent to participate in this study was provided by the participants’ legal guardian/next of kin.

## Author Contributions

TH: investigation, data curation, and writing – original draft and review. ML: conceptualization, methodology, investigation, resources, writing – original draft (lab part) and review, and supervision (Genetic Lab). OE: investigation (inflammatory markers) and writing – review and editing. AN: resources, data curation, and writing – review and editing. AM: supervision, project administration (NL), and writing – review and editing. CS: investigation and writing – review and editing. TW: writing – review and editing. RS: investigation, methodology (inflammatory markers), supervision (lab), resources, and writing – review and editing. SZ: conceptualization (inflammatory markers) and writing – review and editing. CM: investigation and writing – review and editing. LB: formal analysis, funding acquisition (NL), and writing – review and editing. SS-G: conceptualization, methodology, data curation, formal analysis, funding acquisition, writing – original draft and review, supervision, and project administration. All authors contributed to the article and approved the submitted version.

## Conflict of Interest

The authors declare that the research was conducted in the absence of any commercial or financial relationships that could be construed as a potential conflict of interest.

## Footnotes

1 https://www.ncbi.nlm.nih.gov/snp/rs41266431#frequency_tab
