# Supplementary material for: Impact of a Gap Junction Protein Alpha 4 Variant on Clinical Disease Phenotype in F508del Homozygous Patients With Cystic Fibrosis
Source: Front Genet. 2020 Oct 28;11:570403. doi: 10.3389/fgene.2020.570403 (PMC7655539; doi:10.3389/fgene.2020.570403)
Supplement: Supplementary file 1 [file Presentation_1.pdf]

Supplement

Supplement* e*Table 1

CHARACTERISTICS OF GERMAN PATIENTS

	rs41266431	
Genotype	G/G (n=74)	(A/G+A/A) (n=24)
	MEAN	
Age (years)	26.6	29.1
SEX (m/f) (n)	(40/34)	(15/9)
P.aeruginosa+ (%)	68	71
BMI *(% pred.)*	25.6	29.4
Diabetes mellitus	32%	29%
FEV1 *(% pred.)*	64.6	69.7
FEF75 *(% pred.)*	45.4	48.8
FVC (%predicted)	76[#]	85.3[#]

m, male; f, female; *P.aeruginosa* +, *Pseudomonas aeruginosa* colonization (intermittent+chronic); FEV1 (% predicted), forced expiratory volume in 1 s in % predicted, FEF75 (% predicted) forced expiratory flow at 75% of the pulmonary volume in % predicted, FVC(% predicted) forced vital capacity in % predicted

# p< 0.038

CHARACTERISTICS OF GERMAN PATIENTS WITH CHRONIC *P. AERUGINOSA* COLONIZATION

	rs41266431	
	Genotype G/G (n=34)	(A/G+A/A) (n=11)
	MEDIAN	
**Age (years)**	**32**	**40**
**SEX (m/f)**	**(22/12)**	**(9/2)**
**Diabetes mellitus**	**17/34**	**3/11**
**BMI *(percentile)***	**15**	**12**
**FEV1 *(% predicted)***	**47***	**68***
**MEF 25 *(% predicted*)**	**13§**	**22§**
**FVC *(% predicted*)**	**68.5&**	**89.5&**

*p<0.090, §p<0.047, &p<0.050

Supplemental eTable 3

Predicted effect of rs41266431*

**predicted effect by HSF**	**ESE finder (v3.0)**[1]	**SFMap (v1.8)**[1]
no impact on splicing	A nucleotide decreases score for SRSF1 (CGGCC**G**T, 4.54885->1.97111) and increases the score for SRSF2 (GGCC**G**TAG, 2.80185->3.22712)	A nucleotide deletes motifs for SF2ASF (CGGCC**G**U, scored 0.932), hnRNPA2B1 (GGCC**G**UAG, scored 0.625) and hnRNPH1 (**G**UAGAG, scored 0.763)

motif nucleotide affected by the A allele of rs41266431 is given in bold and underlined (36)

[1]threshold for: SRSF1, 1.956; SRSF2, 2.388;

(*Akerman M, David-Eden H, Pinter RY, Mandel-Gutfreund Y. A computational approach for genome-wide mapping of splicing factor binding sites. *Genome Biol*, 2009; 10: R30.)

Online data supplement

Methods

Pulmonary function tests

Most patients were observed between 2010-2017 (72%). Reasons for using data earlier than 2018-2016 was therapy with Orkambi or lost to follow-up. For the Dutch cohort only data from 2017 were available.

Results

Genotyping (for rs41266431) for was available from all patients. Of the 116 patients, 84 were homozygous for the G variant (G/G genotype), 30 heterozygous (A/G genotype) and 2 homozygous for the A variant (A/A genotype). The allele frequency was 0.853 for the G allele and 0.147 for the A allele.

Mortality

There was no significant difference (p<0.531) in observation time between the Dutch and the German cohort (median 26/27.5 y, IQR 22.5-28.75/19-40.25, range 19-45/9-62).

Body mass index (BMI)

There was no significant difference in body mass index (% percentile) for the two genotypes (G/G genotype/ carriers of the A allel, median 23.5/29, CI 23.9-35.64/26.22-50.94, range 0-99/0-100, p<0.197).

Pseudomonas aeruginosa

For genotype I (n=84) 30 (36%) never were colonized with *P.aeruginosa*, 15 (18%) had intermittent and 39 (46%) chronic colonization. In carriers of the A allele (n=31) 9 (29 %) never were colonized, 4 (13%) had intermittent and 18 (58%) chronic colonization.

German patients (n=98)

For the G/G genotype 35/74 (47%) individuals had chronic colonization, 15 (20%) intermittent and 24/74 (32%) were never colonized. For the 24 carriers of the A allele 13/24 (54%) had chronic, 4/24 (17%) had intermittent colonization. Seven of 24 individuals (29 %) never got colonized. There were no significant (p<0.765) differences between the two genotypes for any colonization (68%/71%) vs. never (32.4%/29%).

Dutch (Amsterdam)

For the G/G genotype 4/10 (40%) individuals were chronically colonized,and 6/10 (60%) were never colonized. For the 7 carriers of the A allele 5/7 (71%) had chronic colonization. 2 of 7 individuals (29 %) never got colonized. There were no significant (p<0.201) differences between the two genotypes for chronic colonization vs. never.

Pulmonary function

Overall, there was no significant difference in FEV1% predicted (genotype G/G/(A/G+A/A)): 95% CI 59.8-71.7/63.2-78.1, range 19-129/22-111, p<0.301.

For FVC% predicted there were significant better values (p<0.040) in the carriers of the A allele: 95% CI 73.1-83.2/80.3-92.2, range 29-126/51-119.5).

There was no significant (p<0.356) difference for FEF75% predicted (G/G genotype/(A/G+A/A):, 95% CI 43.2-64.4/44.1-82.6, range 7-203/9-220.

German cohort (n=98)

There was no significant difference in FEV1 (%predicted) (genotype G/G vs (A/G+A/A): CI 60.6-73.6/60.4-79, p<0.332) and FEF75 (%predicted) values (genotype I/II): (genotype G/G vs (A/G+A/A): CI 36.1-54.6/ 30-67.6, p<0.715). For FVC% predicted overall there were significantly (p<0.038) better values for the carriers of the A allel (genotype G/G vs (A/G+A/A)): CI 72.7-83.3/78.3-92.4).

Amsterdam (n=18)

There was no significant difference in FEV1 (%predicted) (genotype G/G vs (A/G+A/A):, IQR 57.3-96.3/51-91.5, p<0.965), FEF75 (%predicted) (genotype G/G vs (A/G+A/A):, IQR 62.1-174/49.5-155, p<0.846) as well as for FVC ((%predicted) (genotype G/G vs (A/G+A/A): IQR 73.3-111.8/75.8-101.8, p<0.745).

Matched pairs

Overall 44 patients (22 per each genotype) were matched by sex and age (+/-2y). There was a trend for a significant difference ($p<0.097$) in FEV1 (%predicted) (G/G Genotype vs carriers of the A allel: median 48 /68, IQR 34-75.5/52.5-80.25, range 19-96/22-111). For FVC (% predicted) (Genotype G/G vs (A/G+A/A): median 65/84, IQR 57-87.75/69-96, range 43-99/51-106, $p< 0.012$) and FEF75 (%predicted) values (Genotype G/G vs (A/G+A/A): median 20/45.5, IQR 17-43.5/21-61, range 7-77/9-174, $p<0.031$) there was a significant difference between the two genotypes.
